# Morphological and Morphometrical Aspects of the Auditory Ossicles in the European Badger (Meles Meles)

**DOI:** 10.3390/vetsci9090483

**Published:** 2022-09-08

**Authors:** Cristian Martonos, Alexandru Gudea, Călin Lațiu, Milos Blagojevic, Florin Stan

**Affiliations:** 1 Faculty of Veterinary Medicine, University of Agricultural Sciences and Veterinary Medicine Cluj-Napoca, Calea Mănăştur 3-5, 400372 Cluj-Napoca, Romania; 2 Faculty of Animal Breeding and Bitotechnologies, University of Agricultural Sciences and Veterinary Medicine Cluj-Napoca, Calea Mănăştur 3-5, 400372 Cluj-Napoca, Romania; 3 Faculty of Veterinary Medicine, University of Belgrade, 1 Studentski Trg, 11000 Belgrade, Serbia

**Keywords:** middle ear, ear ossicles, badger, morphometry, malleus, incus, stapes

## Abstract

**Simple Summary:**

The little-described morphology of the ear ossicles in the badger provides some interesting morphological features alongside some metrical data. For the malleus, we notice the standard framing into the known shape, with the mentioned presence at the level of the column of all three processes (lateral, rostral and medial), from which the rostral one is the most developed. The malleal manubrium is long and triangularly shaped on a cross-section. For the incus we notice the overall shape of a biradicular molar with the existence of the two crura in acute angulation, while the long crus is continuing with the lenticular process. The presence of a bony blade that links to the lenticular process is also noted. For the stapes, the almost equal two crura and the quite round intercrural foramen is described.

**Abstract:**

Given the scarce morphological data regarding the middle ear anatomy of this species, the paper attempts to describe the morphological and morphometrical data of the auditory ossicles in the badger. The study was performed using the standard morphological investigations and provides a complete morphological description of the ossicular assembly (malleus, incus and stapes) with some comparative features and attempts to provide a complete set of standardized metrical data for each ossicle. A more-detailed attempt to compare some functional aspects in the light of combined metrical ratios is also implied.

## 1. Introduction

Taxonomically the badger belongs to Mammalia class, order Carnivora and the Mustelidae family [1].

Studies point to the existence of four subfamilies—Melinae, Mellivorinae, Taxidiinae and Hellictidinae within this larger family. The European badger is framed into the Melinae family, close to the hog badger (*Arctonyx collaris*), which is listed as a species in extinction. The Mellivorinae subfamily includes the melivore badger (*Mellivora capensis*), the Taxidiinae includes the American badger (*Taxidea taxus*) and the *Hellictidinae* includes three *Melogale species* [2].

According to literature sources, the Mustelidae family is the most representative, as far as the number of species is concerned, the family of the Carnivora order, including 67 species spread all over the world, except for Australia and Antarctica [3]. In Europe, the badger is spread over all areas, from forested regions to intensely urbanized areas [4,5]. In regards to their interaction with other species, the European badger is considered a reservoir for *Mycobacterium bovis* in the UK, Ireland, Spain and France [6] and they can play a role as a host for the Trichinella species [3] or *Thelazia callipaeda* [7]. 

The tympanic cavity is described as an air-filled osseous cavity carved into the temporal bone placed between the external ear and the internal ear [8,9]. Available studies on the ear of the badger focus mainly on the anatomy of the cavity of the middle ear and the appearance of the bulla tympanica or other cranial aspects related to its morphology [10].

The tympanic cavity or the middle ear (*Auris media*) is laterally delimited by the tympanic membrane to the oval window medially (*Fenestra vestibuli*). The auditory ossicles are related to this segment as they link the aforementioned parts in an articulated fashion, playing an important role in the transmission and modulation of soundwaves, as described by all morphology sources: the malleus, the incus and the stapes [8,9,11,12,13].

In most mammals, the auditory ossicles appear as individualized pieces, unlike some rodents where the malleus and incus is fused, forming an maleo-incal complex [8,12,14,15,16,17], as in some birds there is only one single bony piece described—*Collumela auris*—that is the equivalent of the stapes in mammals [11,18,19]. For carnivores, a relatively reduced number of species have been studied in this respect, such as the wolf [18], the dog [19,20], the cat and the fox [20,21].

The present study aims to provide a series of morphological and morphometrical data concerning the auditory ossicles in the badger (*Meles meles*) that might be helpful for researchers and clinicians. Some attention is paid to some mechanical and physical elements of the anatomy of the middle ear, specifically pointing at explanatory elements regarding the involvement and correlations of the metrical features of the ear ossicles to the specificity of sound transmission for this wild species, given the importance of this so-called “transitional type” of the ear, situated between the freely mobile and the malleus-incus microtype [22].

The data that support the findings of this study are available from the corresponding author, A.G, upon reasonable request.

## 2. Materials and Methods

The biological material was represented by 5 badger crania originating mainly from road causalities presented in our Emergency Clinic or Pathology Department. The cephalic extremities were subjected to a mechanical cleaning process, after a careful inspection of the temporal bone part, by means of a warm maceration procedure for 3–5 days. After another cleaning procedure, the tympanic bulla was assessed, and several fragmentation actions were performed for the removal of the auditory ossicles from the tympanic cavity. The pieces were carefully identified, cleaned, and assessed from a morphological and morphometrical perspective in the Anatomy Lab of the Faculty of Veterinary Medicine. Usual anatomical and surgical tools were used (tweezers, scalpel and bone rongeur). Digital images were collected (Olympos MTX stereomicroscope and camera) while images were further processed with a Wacom Intuos tablet, Corel Painter Essentials 5 suite. For the measurements of the ossicles, a scaled surface was placed under the specimens before image capturing. ImageJ was the software solution used for the measurements and further numerical processing.

Measurements on the auditory ossicles were made following data suggested by a series of authors [14,18,23,24,25], with some adaptations, as some of the reference points suggested by the available literature were not applicable to our specimens. 

The following measurements were performed:

- LM—length of malleus
- WHM—width of head of malleus
- LhM—length of the handle of malleus
- Li—length of incus
- LSC—length of short crus of incus
- LLC—length of long crus of incus
- HBI—height of the body of incudis
- WBI—width of the body of incudis
- LS—length of stapes
- WHS—width of head of stapes
- WBS—width of base of stapes
- LCC—length of caudal crus of stapes
- LRC—length of rostral crus of stapes
- APd—antero-posterior diameter of obturator foramen of stapes
- DVd—dorso-ventral diameter of obturator foramen of stapes

Depending on the availability of measurements on the digitally collected images for our available biological samples, we tried to establish a minimum number of 6–10 measurements for each item, ensuring statistical representativity. Statistical interpretation was conducted using the basic statistical features of Google sheets formulas (counting, average and standard deviation).

## 3. Results

In the cavity of the middle ear, we identified the assembly of the auditory ossicles represented by the malleus (*Malleus*), incus (*Incus*) and the stapes (*Stapes*).

**Figure 1:**
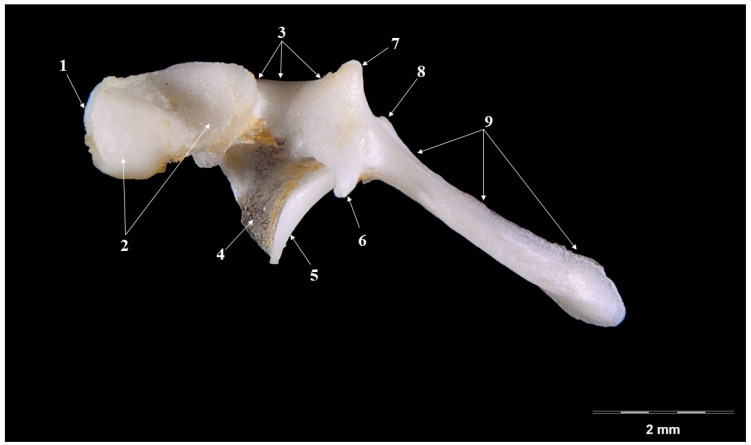
**Malleus-Medial perspective.** 1. Caput mallei; 2. Processus articularis; 3. Collum mallei; 4. Ossicular lamina; 5. Process rostral; 6. Processus muscularis; 7. Processus lateralis—the upper section; 8. Processus lateralis—the lower section; 9. Manubrium mallei.

**Figure 2:**
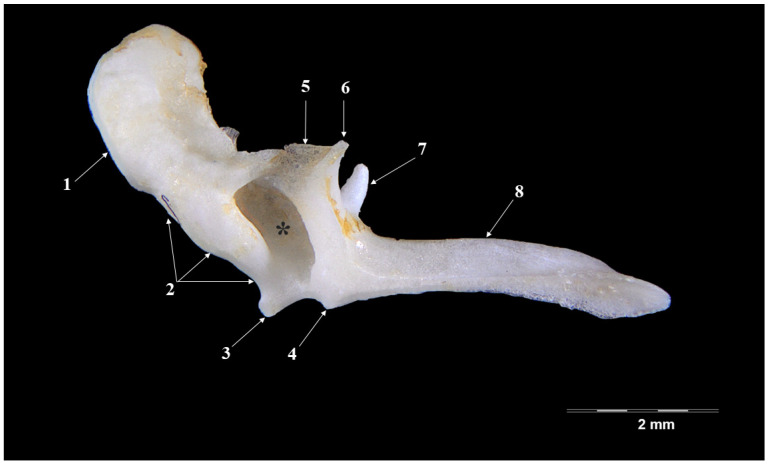
**Malleus-lateral perspective.** 1. Caput mallei; 2. Collum mallei; 3. Processus lateralis—upper segment; 4. Processus lateralis, lower segment; 5. Lamina; 6. Processus rostralis; 7. Processus muscularis; 8. Manubrium mallei; *—innominate fossa.

The most-developed piece is the malleus (Figure 1 and Figure 2). Laterally placed, in contact with the tympanic membrane, it appears slightly curved piece that has three distinctive anatomical parts: the head of malleus (*Caput mallei*), the neck of malleus (*Collum mallei*) and the handle (*Manubrium mallei*).

The head of malleus is slightly ovoidal in shape and it presents two parts—a lateral one (oval, slightly irregulated as surface) and a medial one (with the articular surface for the incus). The articular incal surface with a medio-caudal orientation displays two portions: a dorsal and a ventral one, forming an obtuse angle.

The neck of the malleus continues in an oral-ventral direction to the head of the ossicle. This segment seems quite short, with a series of processes placed very closely, one to another. A rostral process (*Processus rostralis*), the most developed one, detaches from the oral-ventral part of the neck, and appears as an almost-cylindrical pointed bone piece linked to the ventral part of the neck by a fine osseous blade. The muscular process (*Processus muscularis*) is a long piece (Table 1) that detaches from the medio-caudal part of the neck. This process serves as insertion point for the tensor muscle of the tympanic membrane (*M. tensor tympani*). The lateral process (*Processus lateralis*) appears as two small tuberosities separated by a depression onto the dorsal part of the neck, close to the junction of the neck (*collum*) with the handle (*manubrium*).

The handle of malleus continues the neck as a ventral projection towards the tympanic membrane. It is an elongated (Table 1), triangular in transversal section piece, showing a lateral basal part and a tip medially. In its distal part, the manubrium becomes flattered and curves medio-laterally. An overall observation indicates a relatively reduced angulation for the manubrium and neck of the malleus, a fact that makes it comparable to the overall aspect of a hockey stick.

**Figure 3:**
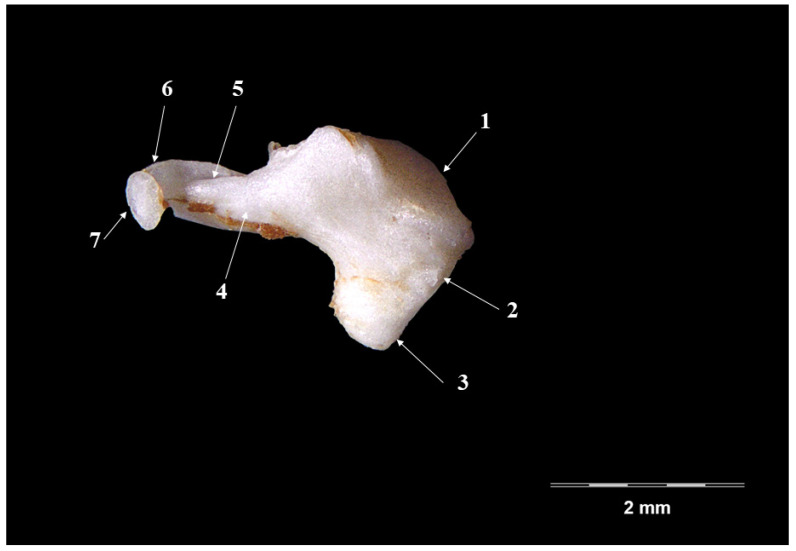
**Incus-medial perspective.** 1. Incundomalearis articular surface, 2. Corpus incundis; 3. Crus breve; 4. Crus longum; 5. Junction point- crus-blade; 6. Bony blade; 7. Processus lenticularis.

**Figure 4:**
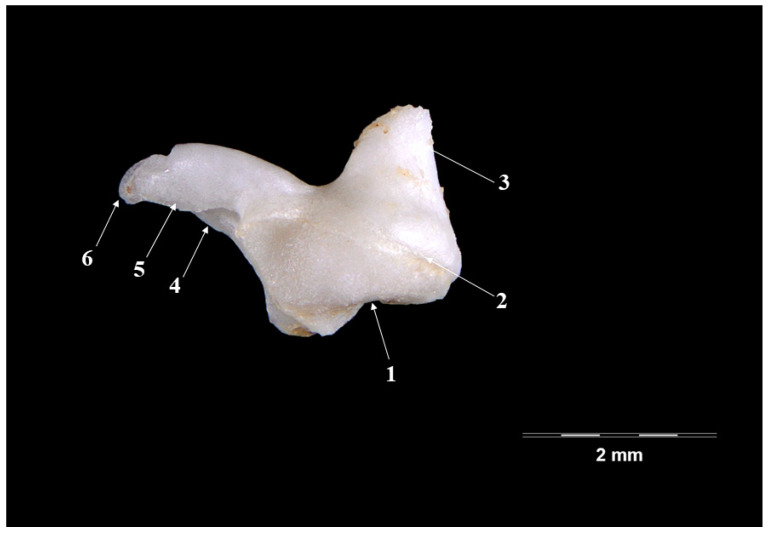
**Incus-lateral perspective.** 1. Incundomalearis articular surface; 2. Corpus incundis; 3. Crus breve; 4. Crus longum; 5. Bony blade; 6. Processus lenticularis.

The incus (Figure 3 and Figure 4), smaller than the malleus (Table 1), continues from the malleus and has the overall aspect of a biradicular molar. Onto its surface, a series of morphological segments can be described and identified: the body (*Corpus incudis*), the short crus (*Crus breve*) and the long crus (*Crus longum*).

The body of the incus appears as a well-developed piece, wider than long, with an evident articular malleal surface (*articulatio incundomallearis*) on its anterior extremity. From its caudal part, the two crura projects as slightly divergent processes, giving the extremity the shape of V letter. The short crus (*crus breve*), shorter and tronconic-shaped, continues to the dorsal margin of the ossicle in a dorso-caudal direction. The long crus, in a more-acute angle, continues to the ventral margin of the ossicle, with a long, pointed or effilated shape (see Table 1).

This terminal part of the process shows, in all investigated specimens, the presence of a quite well-individualized bony blade that continues with the so-called lenticulate process (*Processus lenticularis*). This piece ensures the junction with the third ossicle, the stapes. Based on the relatively good separation of this last part, we can state the fact that in this case, we can describe a really distinct lenticulate piece.

**Figure 5:**
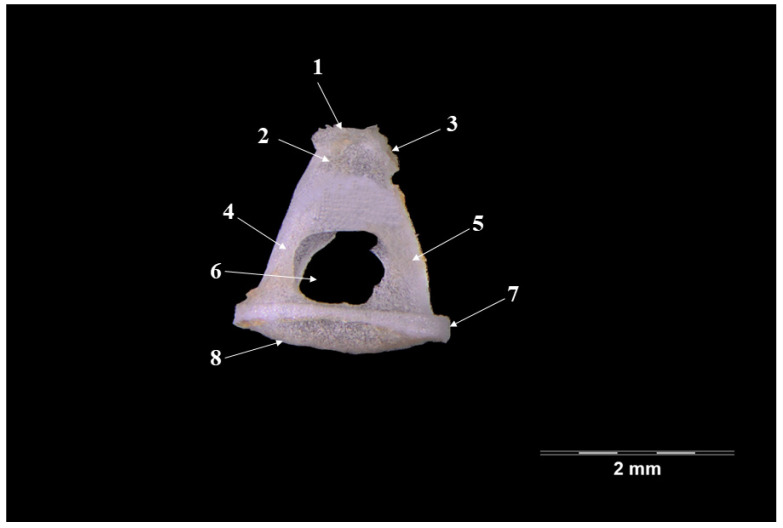
**Stapes-Ventral pespective.** 1. Articulatio incundomallearis; 2. Caput stapedis; 3. Tuberositas m.stapedius; 4. Crus rostrale; 5. Crus caudale; 6. Intercrural foramen; 7. Basis stapedis; 8. Facies interna basis stepedis.

The stapes (Figure 5) is the smallest of the auditory ossicles (Table 1) placed between the lenticular piece and the oval window (*Fenestra vestibuli*). The overall shape of the ossicle is almost triangular, with an oblique, medio-caudal placement. The ossicle has a head (*Caput stapedis*), an rostral crus (Crus rostrale), a posterior crus (*Crus caudale*) and a base (*Basis stapedis*). The smallest of the parts is the head of the stapes. On its proximal part, the presence of the articular surface for the lenticulate piece is visible (*articulatio incundostapedialis*) while not far from its caudal part another rough surface was visible—the insertion point for the stapedial muscle (*m.stapedius*).

Distally, the ossicle continues with the two crura that link to the basal part (*Basis stapedis*). The rostral crus appear thinner and almost straight, while the caudal one is thicker at its base and slightly curved. The boundary of the almost circular intercrural orifice (*foramen intercrurale*) is the space between the two crura. The base of the stapes has an elliptical shape, with a quite accentuated convexity that fits the oval window (*fenestra vestibuli*).

## 4. Discussion

Otology studies rely greatly on animal models. One of the most-frequently used animal models is represented by small rodents but other mammalian species are often referred to as interesting species due to their resemblance with the ossicular assembly (functional and morphological) in primates and humans [25,26,27,28,29,30]. The middle ear, in its morphology, can be regarded as a pressure amplifier. The ossicular arrangement, its joints, ligaments and muscles do nothing but change the efficiency of the sound transmission [28,31,32,33,34] and generate a mechanical advantage, from the tympanic membrane to the oval window towards the cochlear system.

As in most mammals, the ossicular auditory assembly is comprised of the standard three sets of entities: the malleus, the incus and the stapes. In some exceptional cases, literature sources name the fourth ossicular component—the lenticular bone—as a separate, individualized bone piece [35,36,37,38] in some donkey, Indian mongoose and human individuals. 

In some rodents, there is another peculiar situation that points to a certain physiological degree of fusion for the first two ossicles, forming the maleo-incal complex, such as in the Guinea pig [39,40], paca [16], degu [41], the hamster [12] or chinchilla [8,14]. In some other situations, the fusion of these ossicles (ossicular fixation) is cited as correlating with a loss of hearing, such as in humans and mice [42,43,44].

The overall shape of the head of the malleus in the badger (*Meles meles*) (Figure 1 and Figure 2) seems similar to the description in other mammals [11,45]. In some caviomorph rodents, the head of malleus is described as “bullet-shaped” [41]. The neck of the malleus displays three processes: the rostral one, the lateral one and the muscular one. In the small Indian mongoose (*Herpetes Javanicus*), the muscular process, unlike some other carnivores, detaches from the internal margin of the handle of the malleus [36]. A well-developed muscular process is cited in donkeys, foxes and cats [20,22,23]. In badgers, the rostral process is the most-developed, similar to that of domestic goats and leporids [11,12], while for some rodent species this process is very reduced or even absent [46]. The handle of the malleus (Table 1) in the badger has a triangular shape on a cross-section, similar to that reported in the goat. In other species, such as the buffalo, cow and sheep, this section is almost quadrilateral [13]. A special note is made for the small Indian mongoose, where the authors described at the base of the handle of malleus a visible transversal groove that supports the passage of the chorda tympany nervous branch [36].

The incus (Figure 3 and Figure 4) in the badger has the well-known aspect of a biradicular molar [11,13,20]. The two divergent processes of the ossicle seem to be placed in a more acute angulation when compared with that described in the rabbit and the hamster [24], where these processes seem almost perpendicular to one another. As far as their size is concerned (Table 1), the long crus is much longer than the short one, which is similar to that described in other carnivores [36]. The relatively similar length of the two processes is cited in a series of small [11,47] or large ruminants [13]. 

The direct continuation of the long crus with the lenticulate process has been cited in many mammalian species. For the badger, the existence of a bony blade that links to the lenticulate piece to the articular surface with the incus makes the statement about the lenticular piece being an individualized piece quite valid. The same stands for the situation encountered in the case of the *Herpetes Javanicus* (small asian mongoose) or the cat [36,48] or even for some young mouse and hamster individuals or humans [8,39,40]. In contrast, the lack of this piece in the case of sheep fetuses has to be cited [45].

The anatomo-topographical disposition of the stapes (Figure 5) in the badger follows the literature data for many species. The triangular-trapezoidal overall shape of the piece has been cited in the wolf [18], domestic goat [11], guinea pig [49], chinchilla [50] and the hamster [51]. Different morphological aspects were cited in some ruminant species, where the incus is framed into a rectangular shape [11,13,20,47] or an irregular round shape (as in the rat [49]). The presence of the insertion surface for the stapedial muscle has been noted in humans [52,53] and sheep fetuses [45], while in other species, only a small tubercle is mentioned, such as in the wolf [18], dog [19], or the buffalo [13]. The overall length for the two processes of the stapes seems equal (Table 1), similar to the one described in swine [45,54], camels and donkeys [35]. The intercrural foramen maintains the overall round shape, while in the chinchilla, this space is more elliptic due to its longer diameter.

One of the most-frequent relations established for the theories of sound transmission in many studies is the isometric relationship between the eardrum area (tympanic area) and the oval window area (A1:A2 ratio) and the ratios of the malleus and incus, mainly the lever arm lengths I 1 and I2 for the malleus and incus that are important in the transformer ratios of the middle ear [20,55]. The malleus and the incus can be regarded as a type 1 lever that has a counter-clock rotational movement as the tympanic membrane moves inward, pressing then against the internal crus of the stapes onto the oval window, with a cited ratio up to 19:1 effective tympanum to oval window [33,56,57,58]. Overall, the middle ear seems to play a highly complex role in sound modulation, dependent not only on these aforementioned factors, the air volumes, stiffness of the tympanic membrane and ossicular system alongside some other non-ossicular sound conduction influences being other secondary factors that make the understanding of the phenomena even more complex [58]. 

In this perspective, a slightly simpler comparison of the main metrical data for the ossicular assembly (as part of the main transmission system) of the some of the available metrical data pointing to middle ear ossicles can be easily illustrative for the adaptative changes in ear morphology, but not minimizing the importance of the other physical properties mentioned earlier.

This series of ratios were calculated based on the available literature data [20,22,25] between different lengths of the ossicular assembly in carnivores, ruminants and non-ruminants and some rodents [8,11,20,50,51,55,59].

**Figure 6:**
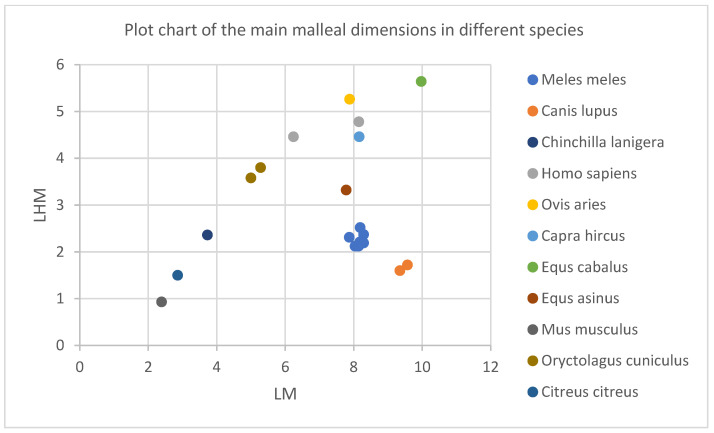
Graphical representation of the malleal ratios for different species.

**Figure 7:**
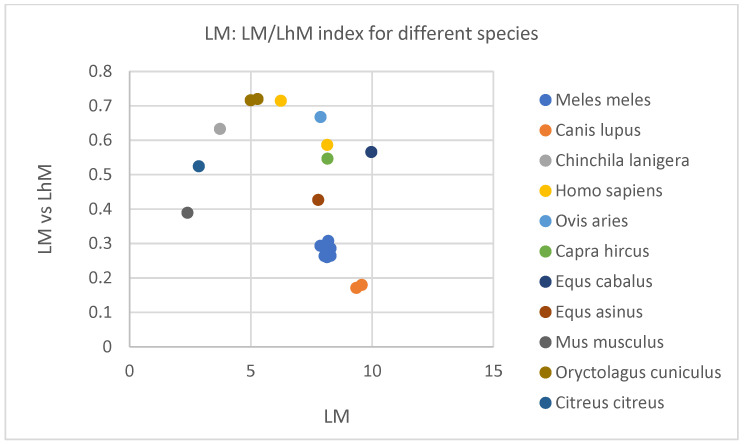
Graphical representation of the malleal length vs. the index calculated as a fraction of LM from LhM for different species.

**Figure 8:**
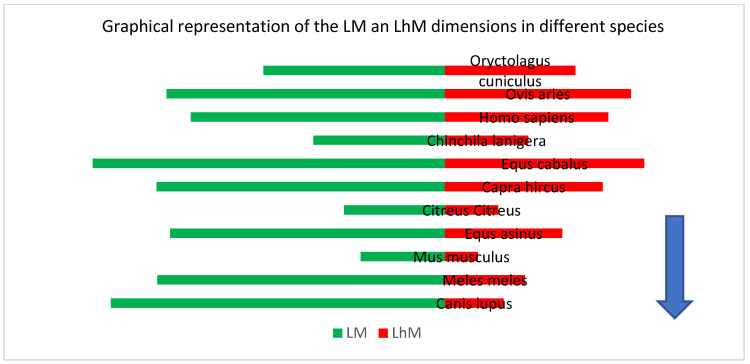
Representation of the malleal proportions in order of the LM:Lhm ratio (descending).

The simple graphical representations (Figure 6) point to some expected morphological aspects: the overall dimensions for such differently sized species places the large species in the most upper part of the graph (horse). On the other hand, in the upper and middle registry, the dimensions for humans alongside the ones for small ruminants seems to be somehow grouped, while the small-sized species (the rodents) occupy the lower registry of the graph. When the graph (Figure 7) takes into consideration the index calculated as fractions of the angle length vs. the overall length of the malleus, one can see the placement of the carnivores in the lowest part of the graph, with the data from humans and some rodents in the uppermost part of the registry. Once the same ratio of the main malleal dimensions are represented as a bar chart, based on the value represented by the proportion of the length of the manubrium from the greatest length of the malleus, an interesting placement appears (Figure 8).

This representation places on the upper part of the chart values for the malleus of some rodents along with those of humans, small ruminants and equids, while the ones from carnivores (wolf and badger) are placed in the lower part of this chart. Such a situation may suggest some kind of correlation with the facts linked to the frequency ranges that are associated with different species’ audible spectrum [60] that points to a higher frequency for mice (up to 91,000 Hz), carnivores (up to 64,000 Hz) and much lower ones for ruminants (sheep) or non-ruminants (horse) (up to 30,000 Hz) or even chinchillas and humans (20,000–22,000 Hz).

**Figure 9:**
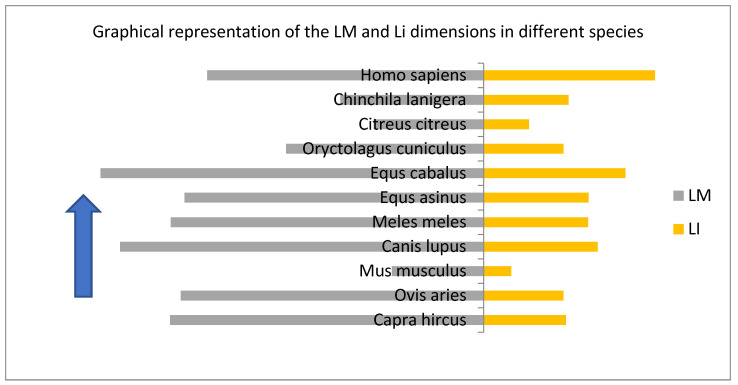
Representation of the malleus:incus proportions (ascending).

The widely used ratio of malleus:incus length ratio for the same series of species (Figure 9) reveals another interesting situation.

One can state a series of similar figures for all species (Table 2) for the proportions of the malleus (ratio lever:malleus), while for the calculations that imply incus dimensions, figures for humans stand aside from those calculated in carnivores (ratio malleus:incus). Similar asymmetry can be observed in case of ratios malleus:stapes and the short crural length vs. the stapes dimensions, where the humans seem to have another value, slightly different to the ones of carnivores. These data may serve again as a differential adaptative difference among those species, with a direct connection to physiological adaptative changes related to environmental factors and evolution.

## 5. Conclusions

The present study, to the best of our knowledge, brings into light some new elements of the middle ear anatomy, with respect to the ossicular anatomy, for a species relatively little-studied. This is the main purpose of the study—the complete morphological description of the ear ossicles in the badger, alongside some morphometrical data. These, combined with some graphical elements, are meant to serve as a useful didactical tool in the study of the comparative morphology of the middle ear and to point to some new directions in this study.

## Figures and Tables

**Table 1:** Metrical data for the auditory ossicles in the badger (*Meles meles*).

Measurement	Average Value (mm)	No of Measurements
**Malleus**		
**LM**	8.142 ± 0.14	9
**LHM**	2.264 ± 0.014	8
**WHD** ***(not to include the bony lamellae)***	1.57 ± 0.06	7
**LhM**	4.854 ± 0.15	8
**Incus**		
**LI**	2.72 ± 0.12	5
**LSC**	1.995 ± 0.1	6
**LLC**	2.69 ± 0.21	8
**HBI**	1.48	3
**WBI**	2.075 ± 0.14	5
**Stapes**		
**WBS**	2.16	3
**LCC**	2.095 ± 0.2	5
**LRC**	1.95 ± 0.11	6
**WHS**	0.79 ± 0.13	6
**LS**	2.3 ± 0.08	7
**Apd**	0.91	2
**DVd**	0.81	2

**Table 2:** Comparison of combined numerical data for the badger, wolf and humans.

	*Meles meles*	*Canis lupus*	*Homo sapiens*	*Ovis aries*	*Capra hircus*
*Ratio lever/malleus*	*1:0.59*	*1:0.74*	*1:0.586*	*1:0667*	*1:0.5465*
*Ratio maleus/incus*	*1:0.334*	*1:0.309*	*1:0.662*	*1:0.305*	*1:0.262*
*Ratio maleus/stapes*	*1:0.278*	*1:0.272*	*1:0.402*		
*Ratio L lc/H stapes*	*1:1.18*	*1:1.07*	*1:1.6*		

## Data Availability

Not applicable.
